# Phylogenetic study and taxonomic revision of the *Xanthoparmeliamexicana* group, including the description of a new species (Parmeliaceae, Ascomycota)

**DOI:** 10.3897/mycokeys.40.26724

**Published:** 2018-09-18

**Authors:** Alejandrina Barcenas-Peña, Steven D. Leavitt, Jen-Pan Huang, Felix Grewe, H. Thorsten Lumbsch

**Affiliations:** 1 Science & Education, The Field Museum, 1400 South Lake Shore Drive, Chicago, IL 60605-2496, USA The Field Museum Chicago United States of America; 2 Department of Biology and M.L. Bean Life Science Museum, Brigham Young University, 4102 Life Science Building, Provo, UT 84602, USA Brigham Young University Provo United States of America

**Keywords:** Cryptic species, lichenised fungi, Mexico, phylogeny, taxonomy

## Abstract

*Xanthoparmelia* (Parmeliaceae, Ascomycota) is the most species-rich genus of lichen-forming fungi. Species boundaries are based on morphological and chemical features, varying reproductive strategies and, more recently, molecular sequence data. The isidiate *Xanthoparmeliamexicana* group is common in arid regions of North and Central America and includes a range of morphological variation and variable secondary metabolites – salazinic or stictic acids mainly. In order to better understand the evolutionary history of this group and potential taxonomic implications, a molecular phylogeny representing 58 ingroup samples was reconstructed using four loci, including ITS, mtSSU, nuLSU rDNA and MCM7. Results indicate the existence of multiple, distinct lineages phenotypically agreeing with *X.mexicana.* One of these isidiate, salazinic acid-containing lineages is described here as a new species, *X.pedregalensis* sp. nov., including populations from xerophytic scrub vegetation in Pedregal de San Angel, Mexico City. *X.mexicana* s. str. is less isidiate than *X.pedregalensis* and has salazinic and consalazinic acid, occasionally with norstictic acid; whereas *X.pedregalensis* contains salazinic and norstictic acids and an unknown substance. Samples from the Old World, morphologically agreeing with *X.mexicana*, are only distantly related to *X.mexicana* s. str. Our results indicate that *X.mexicana* is likely less common than previously assumed and ongoing taxonomic revisions are required for isidiate *Xanthoparmelia* species.

## Introduction

The family *Parmeliaceae* is the largest family of lichenised fungi (Jaklitsch et al. 2016) currently classified in approximately 70 genera with almost 2,800 species (Lumbsch and Huhndorf 2010, Divakar et al. 2017). *Xanthoparmelia*, with about 800 described species, is the largest genus of lichen-forming fungi (Lücking et al. 2016), with two centres of distribution in Australia and southern Africa; a smaller number of species occur in the Holarctic (Blanco et al. 2004, Eriksson et al. 2004, Crespo et al. 2010, Thell et al. 2012, Leavitt et al. 2018). To date, 74 species have been reported from Mexico, amongst these species, 27 are isidiate (Nash et al. 2016).

Isidiate *Xanthoparmelia* species are distributed in boreal, temperate and tropical regions. However, they commonly occur in semi-arid to arid regions worldwide especially on siliceous rocks, such as granite and sandstone. In North and Central America, *Xanthoparmeliamexicana* (Gyelnik) Hale ranks amongst the most common isidiate species. This taxon is widely distributed and has been reported from western USA, Mexico, Dominican Republic, Argentina, Kenya, Australia, New Zealand, Japan, China and Nepal (Hale 1990, Elix 1994, Nash and Elix 2004). *X.mexicana* is part of a complex of morphologically similar species, with adnate to slightly attached thalli, cylindrical isidia and a brown lower side of the thalli, which are primarily separated by their secondary metabolites. The species complex also includes *X.ajoensis* (T. H. Nash) Egan (diffractaic acid), *X.dierythra* (Hale) Hale (norstictic acid), *X.joranadia* (T. H. Nash) Hale (lecanoric acid), *X.maricopensis* T. H. Nash & Elix (norstictic and hyposalazinic acids), *X.moctezumensis* T. H. Nash (3-α-hydroxybarbatic acid), *X.plittii* (Gyelnik) Hale (stictic acid), *X.schmidtii* Hale (barbatic, norstictic and salazinic acids), *X.subramigera* (Gyelnik) Hale (fumarprotocetraric acid) and *X.weberi* (Hale) Hale (hypoprotocetraric acid) (Hale 1990, Nash et al. 2016). However, previous studies indicate that current interpretations of morphological features and secondary metabolites likely fail to accurately characterise species-level diversity in isidiate *Xanthoparmelia* species (Leavitt et al. 2011, 2013).

To better understand the evolutionary history of the *Xanthoparmeliamexicana* complex and potential taxonomic implications, isidiate *Xanthoparmelia* specimens were collected from different locations throughout arid regions of Mexico and supplemented with previously available sequence data. The new samples came from xerophytic scrublands in the states Puebla, Oaxaca, San Luis Potosí, Querétaro, Estado de México, Mexico City, Guanajuato, Zacatecas and Hidalgo, all in the central part of Mexico. We focused on sampling *Xanthoparmelia* populations that were phenotypically similar to *X.mexicana*, e.g. isidiate specimens containing salazinic acid. *X.mexicana* was originally described by Gyelnik (1931) as *Parmeliamexicana* and was later combined into *Xanthoparmelia* by Hale (1974). The type specimen was collected from San Jerónimo, in Pedregal de San Angel, Mexico City. The syntype in the Bouly de Lesdain herbarium was destroyed during World War II, whereas the lectotype in the Budapest herbarium (BP) was not available for molecular study. Therefore, we attempted to recollect material at the type locality of *X.mexicana* and other regions throughout Mexico. Based on the results of this study, we formally describe a previously unrecognised species-level lineage comprised of isidiate specimens as new to science.

## Material and methods

### Taxon sampling

**Figure 1.**
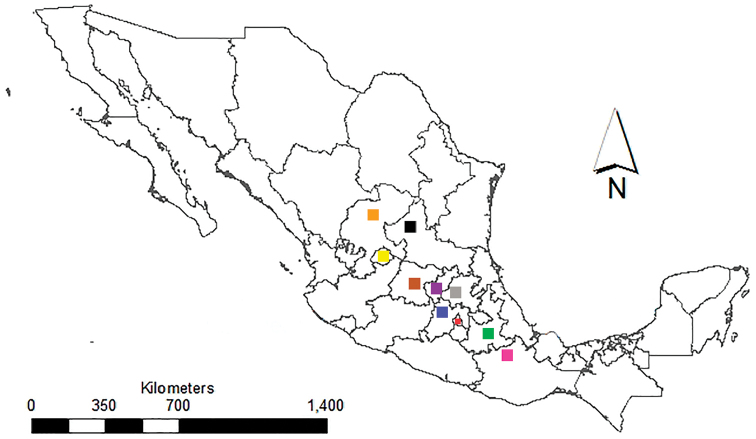
Location of new *Xanthoparmelia* recollection sites from arid regions from central part of Mexico. Oaxaca (pink), Puebla (green), Mexico City (red), Estado de México (blue), Querétaro (purple), Guanajuato (brown), Hidalgo (grey), Aguas Calientes (yellow), San Luis Potosí (black), Zacatecas (orange).

Specimens were studied from the herbaria ASU, BRY, F, MAF and new collections from different localities throughout arid regions from the central part of Mexico (Table 1, Fig. 1). A total of 83 specimens, representing 43 species were included, with an emphasis on isidiate species/populations from Central and North America (all epithets are validly published, with the exception of *X.isidiomontana nom prov* assigned to the clade ‘D2’ from Leavitt et al. 2013). New sequences were generated from 25 specimens and supplemented with 34 sequences from a previous analysis (Leavitt et al. 2018) and 24 additional sequences from GenBank (Table 1). Four species in the genus *Xanthoparmelia* that have previously been shown to be distantly related to *X.mexicana* were used as outgroup – *X.beatricea*, *X.austroafricana*, *X.subramigera* and X.aff.subramigera (Leavitt et al. 2018).

**Table 1.** Collection information for specimens included in the present study: Species, morphological/chemical species identification; DNA code, individual code associated with specimen label in multiple sequence alignments; Species distribution; Voucher information; and GenBank accession numbers for sampled loci in bold text indicates new sequences generated for this study. Specimens sequenced using Illumina technology are indicated by a • with the associated DNA code.

Species	DNA code	Voucher	ITS	MCM7	mtSSU	nuLSU
* X. aff. chlorochroa *	082f	USA: Utah; Leavitt et al. 55225 (BRY-C)	MG695498	MG695699	MG695746	MG695599
* X. aff. chlorochroa *	9866	USA: Nevada; Leavitt & St. Clair 9866 (BRY-C)	MG695499	MG695700	MG695747	MG695600
* X. aff. coloradoensis *	135f	USA: Utah; Leavitt et al. 55255 (BRY-C)	MG695500	MG695701	MG695748	MG695601
* X. aff. protomatrae *	GenBank	Spain: Zamora; Blanco & Crespo 6216 (MAF-Lich)	AY581104	–	AY582339	AY578972
* X. aff. subramigera *	9664	Kenya: Coast, Kirika & Lumbsch 4117 (F)	MG695515	–	MG695764	MG695616
*X.ajoensis* •	14908	Mexico: Puebla; Barcenas-Peña 5898 (F)	MH580218	MH686124	MH699893	MH699913
*X.ajoensis* •	14920	Mexico: Puebla; Barcenas-Peña 5900 (F)	MH580219	MH686125	MH699894	MH699914
*X.ajoensis* •	14934	Mexico: Puebla; Barcenas-Peña 5914 (F)	MH580220	MH580220	MH699895	MH699915
* X. angustiphylla *	GenBank	USA: Blanco et al. 6768 (MAF)	AY581092	–	AY582328	–
* X. atticoides *	GenBank	USA: Blanco et al. 6744 (MAF)	AY581066	–	AY582302	AY578929
* X. austroafricana *	9549	Kenya: Coast Prov., Kirika 4485 (F)	MG695542	–	–	MG695644
* X. beatricea *	E467	Kenya: E467 (MAF-Lich 17174)	JQ912367	–	MG695793	JQ912462
*X.camtschadalis* 1	GenBank	USA: Leavitt et al. 55174 (BRY-C)	HM578630	–	–	HM579042
*X.camtschadalis* 2	GenBank	USA: Leavitt et al. 55291 (BRY-C)	HM578744	–	–	HM579156
* X. cf. mexicana *	016m	Pakistan: Tattu; Kahlid, Usman & Khan MKF16 (LAH)	MH580221	–	–	–
* X. cf. mexicana *	016m2	Pakistan: Swat Valley; Khan & Khalid SW-16 (LAH)	MH580222	–	–	–
* X. chlorochroa *	536f	USA: North Dakota; G. Lind 1213 (BRY-C)	HM578887	HM579688	KR995372	HM579298
* X. conspersa *	GenBank	Spain: Zamora, Blanco & Crespo s.n. (MAF-Lich 6793)	AY581096	–	AF351186	AY578962
* X. cordillerana *	E422	Chile: E422 (MAF-Lich 17198)	JQ912358	–	MG695797	JQ912453
*X.coreana* 1	GenBank	South Korea: Hur, J.-S. 005561	KJ170890	–	–	KJ170890
*X.coreana* 2	GenBank	South Korea: Hur, J.-S. 005589	KJ170883	–	–	KJ170883
*X.coreana* 3	GenBank	South Korea: Hur, J.-S. 013905	KJ170873	–	–	KJ170873
* X. cumberlandia *	nybg02	USA: Maine; R. Harris 55563 (NY)	MG695545	–	MG695798	MG695646
* X. dierythra *	226f	USA: Leavitt et al. 55300 (BRY-C)	HM578753	HM579569	–	HM579165
* X. dierythra *	439f	USA: Leavitt et al. 55383 (BRY-C)	HM578833	–	–	HM579245
* X. dierythra *	098f	Mexico: Puebla; Leavitt et al. 55234 (BRY-C)	HM578689	HM579504	–	HM579099
* X. hirolsakiensis *	GenBank	South Korea: Hur, J.-S. 010532	KJ170876	–	–	KJ170876
* X. hypofusca *	8837	USA: West Virginia; Streets (02086946 NY)	MG695550	MG695717	MG695803	MG695651
*X.idahoensis* 1	GenBank	USA: Leavitt et al. 55463 (BRY-C)	HM578915	HM579708	–	HM579323
*X.idahoensis* 2	GenBank	USA: Leavitt et al. 55354 (BRY-C)	HM578805	HM579620	–	HM579216
* X. infrapallida *	9904	USA: Arizona; Leavitt 9904 (BRY-C)	MG695555	MG695720	MG695809	MG695656
* X. isidiovagans *	GenBank	Spain: 9956 (MAF-Lich)	AY581094	JX974718	AY582330	AY578960
* X. lavicola *	GenBank	USA: Leavitt et al. 55230 (BRY-C)	HM578685	HM579500	–	–
* X. lavicola *	15489	Mexico: Morelos; Nash III 46261 (WIS)	MH580227	MH686131	–	MH699920
*X.lavicola* •	14894	Mexico: Puebla; Barcenas-Peña 5857 (F)	MH580223	MH686127	MH699896	MH699916
*X.lavicola* •	14905	Mexico: Puebla; Barcenas-Peña 5884 (F)	MH580224	MH686128	MH699897	MH699917
*X.lavicola* •	14906	Mexico: Oaxaca; Barcenas-Peña 5905 (F)	MH580225	MH686129	MH699898	MH699918
*X.lavicola* •	14910	Mexico: Puebla; Barcenas-Peña 5888 (F)	MH580226	MH686130	MH699899	MH699919
* X. lineola *	245f	USA: Arizona; EA collection 31–259 (55306 BRY-C)	MG695556	MG695721	MG695810	MG695657
* X. maricopensis *	6698	USA: Arizona; J. Leavitt 001 (BRY-C)	MG695558	MG695723	MG695812	MG695659
* X. mexicana *	291f	USA: Leavitt et al. 55328 (BRY-C)	HM578780	HM579596	–	HM579192
* X. mexicana *	786f	USA: Leavitt et al. 55462 (BRY-C)	HM578914	HM579707	–	HM579322
* X. mexicana *	097f	Mexico: Leavitt et al. 55233 (BRY-C)	HM578688	HM579503	-	HM579098
* X. mexicana *	GenBank	South Korea: Jang et al. 005486 (KoLRI)	KM250123	–	–	–
* X. mexicana *	15479	Mexico: San Luis Potosí; Barcenas-Peña 7316 (F)	MH580231	MH686135	MH699904	MH699923
* X. mexicana *	15472	Mexico: San Luis Potosí; Barcenas-Peña 7408 (F)	MH580229	MH699932	–	MH699922
* X. mexicana *	15466	Mexico: San Luis Potosí; Barcenas-Peña 7441 (F)	MH686404	MH686133	MH699902	–
* X. mexicana *	15461	Mexico: Querétaro; Barcenas-Peña 7178 (F)	MH686401	MH699930	MH699901	–
* X. mexicana *	15485	Mexico: Querétaro; Barcenas-Peña 7209 (MEXU)	MH686402	MH686136	MH699905	–
* X. mexicana *	15471	Mexico: San Luis Potosí; Barcenas-Peña 7273 (F)	MH686403	MH699931	MH699903	–
* X. mexicana *	15473	Mexico: Hidalgo; Nash III 45126 (WIS)	MH580230	MH686134	–	–
* X. mexicana *	156f	USA: Leavitt et al. 55267 (BRY-C)	HM578721	HM579536	–	HM579132
* X. mexicana *	15487	Mexico: Hidalgo; Barcenas-Peña 7470 (F)	MH580232	MH686137	MH699906	–
*X.mexicana* •	14899	Mexico: Oaxaca; Barcenas-Peña 5918 (F)	MH580228	MH686132	MH699900	MH699921
*X.moctezumensis* •	14897	Mexico: Puebla; Barcenas-Peña 5891(F)	MH580233	MH686138	MH699907	MH699924
*X.norchlorochoroa* 1	GenBank	USA: Leavitt et al. 55157 (BRY-C)	HM578613	HM579432	–	HM579025
*X.norchlorochoroa* 2	GenBank	USA: Leavitt et al. 55447 (BRY-C)	HM578899	HM579693	–	HM579307
* X. orientalis *	GenBank	South Korea: Hur, J.-S. 005613	KJ170884	–	–	KJ170884
* X. pedregalensis *	527	Mexico: Mexico City; Ruiz-Cazares 1552 (F)	MH580238	MH707353	MH699912	MH699929
* X. pedregalensis *	526	Mexico: Mexico City; Ruiz-Cazares 1553 (MEXU)	MH580234	MH707352	MH699908	MH699925
* X. pedregalensis *	533	Mexico: Mexico City; Ruiz-Cazares 1557 (F)	MH580236	–	MH699910	MH699927
* X. pedregalensis *	529	Mexico: Mexico City; Ruiz-Cazares 1555 (F)	MH580235	MH686139	MH699909	MH699926
* X. pedregalensis *	531	Mexico: Mexico City; Ruiz-Cazares 1559 (MEXU)	MH580237	MH707354	MH699911	MH699928
* X. plittii *	498f	USA: North Carolina; Leavitt et al. (55422 BRY-C)	MG695562	MG695727	–	MG695664
*X.psoromifera* 1	GenBank	USA: Leavitt et al. 55314 (BRY-C)	HM578766	HM579582	–	HM579178
*X.psoromifera* 2	GenBank	USA: Leavitt et al. 55313 (BRY-C)	HM578765	HM579581	–	HM579177
* X. pulvinaris *	GenBank	Hungary: Molnar et al. 93943 (BP)	JQ362484	–	JQ362485	JQ362486
*X.isidiomontana* nom. prov.	292f	USA: Nevada; Leavitt (55329 BRY-C)	MG695579	MG695733	MG695834	MG695679
*X.isidiomontana* nom. prov.	E1010	Spain: E1010 (MAF-Lich 17181)	JQ912354	–	MG695835	JQ912451
*X.isidiomontana* nom. prov.	E984	USA: E984 (MAF-Lich 17199)	JQ912386	–	MG695836	JQ912479
* X. stenophylla *	5040	Kazakhstan: Karkaralinsk; Tshernyshev (BRY-C)	MG695583	MG695737	MG695843	MG695683
* X. stenophylla *	E708	Turkey: E708 (MAF-Lich 17196)	JQ912372	–	MG695844	JQ912467
* X. subcumberlandia *	121f	USA: Utah; Leavitt et al. (55242 BRY-C)	MG695584	MG695738	MG695845	MG695684
*X.subdifluens* 1	GenBank	Spain: de Paz et al. 17178 (MAF-Lich)	JQ912381	–	–	JQ912474
*X.subdifluens* 2	GenBank	Spain: Blanco et al. 9910 (MAF)	AY581105	–	AY582340	AY578973
* X. sublaevis *	GenBank	Spain: Tenerife, Canary Islands; Blanco et al. 7460 (MAF)	AY581106	–	AY582341	AY578974
* X. subramigera *	9668	Kenya: Coast, Kirika 4583 (F)	MG695525	MG695709	MG695774	MG695626
* X. tuberculiformis *	GenBank	South Korea: Jang et al. 012058 (KoLRI)	KM250131	–	–	KM250131
* X. vicentei *	GenBank	Spain: Salamanca; Crespo & Molina (7248 MAF-Lich)	AY581112	–	AY582347	AY578980
*X.viriduloumbrina*1	GenBank	USA: Pennsylvania; Lendemer 13314: 1049917 (NY)	HM066945	–	–	–
*X.viriduloumbrina* 2	GenBank	USA: Pennsylvania; Lendemer 13325: 1049906 (NY)	HM066944	–	–	–
* X. wyomingica *	001f	USA: Utah; Leavitt et al. (55151 BRY-C)	MG695598	MG695745	MG695864	MG695698
* X. wyomingica *	826f	USA: Wyoming; Leavitt 826 (55501 BRY-C)	HM578953	HM579746	–	HM579360

### Morphology and chemistry

Morphological characters were observed using a Zeiss Stemi 2000-C stereoscope. Ascomatal anatomy, ascospore in addition to conidia shape and size were observed using a Zeiss Axioscope. Secondary metabolites were identified using spot test KOH 10%, KC, C, PD and high-performance thin layer chromatography (HPTLC), using solvent systems C following established methods (Culberson and Johnson 1982, Arup et al. 1993, Lumbsch 2002, Orange et al. 2010).

### Molecular methods

Total genomic DNA was extracted from thallus fragments following the manufacturers’ instructions using the ZR Fungal/Bacterial DNA Miniprep Kit (Zymo Research Corp., Irvine, CA). DNA sequences were generated for four markers using polymerase chain reaction (PCR): the nuclear ribosomal internal transcribed spacer region (ITS), a fragment of nuclear large subunit rDNA (nuLSU), the nuclear protein-coding marker minichromosome maintenance complex component 7 (*MCM7*) and a fragment of the mitochondrial small subunit rDNA (mtSSU). PCR reactions contained 6.25 ml of MyTaq Mix, 25 ml H_2_O, 0.25 ml forward and reverse primer and 0.5 ml template DNA, for a total reaction volume of 12.5 ml. The ITS region was amplified using primers ITS1F (Gardes and Bruns 1993) and ITS4 (White et al. 1990); *MCM7* using primers MCM7-709f and Mcm7-1348r (Schmitt et al. 2009), mtSSU using primers mrSSU1 and mrSSU3R (Zoller et al. 1999) and nuLSU rDNA using primers AL2R (Mangold et al. 2008) and LR6 (Vilgalys and Hester 1990). PCR products were sequenced using an ABI PRISM 3730 DNA Analyser (Applied Biosystems) at the Pritzker Laboratory for Molecular Systematics and Evolution at The Field Museum, Chicago, Illinois, USA. Nine specimens were obtained previously for a global phylogenetic study of the genus and sequenced using next generation sequencing technology as described in Leavitt et al. (2018) (Table 1). In short, metagenomic Nextera libraries (prepared from total DNA extraction) were sequenced on the Nextseq platform at the Core Genomics Facility at the University of Illinois at Chicago, USA. Illumina reads of each specimen were mapped to reference marker sequences downloaded from Genbank (ITSAY581063, nuLSUHM125760, MCM7HM579689, mtSSUKR995373) using the mapping feature implemented in Geneious v11.0.3 (http://www.geneious.com, Kearse et al. 2012). The consensus sequence of each locus was extracted and added to the data set of Sanger sequences to build a combined alignment.

### Sequence alignment and phylogenetic analysis

Sanger sequences, consensus Illumina reads and sequences available on GenBank were added to an alignment published in Leavitt et al. (2018) using Mafft v7 with the option ‘add sequence’ (Table 1). ITS, MCM7, mtSSU and nuLSU sequences were aligned independently using the ‘automatic’ option in Mafft v7, with the remaining parameters set to default values. Ambiguous positions of each one-locus alignment were removed using options for a “less stringent” selection on Gblocks 0.91b (Castresana 2000). SequenceMatrix software (Vaidya et al. 2011) was used for the alignment concatenation. Phylogenetic analyses were performed using Maximum Likelihood (ML) and Bayesian Analysis (BA). ML trees were calculated with RAxML-HPC2 on XSEDE 8.2.10 (Stamatakis 2014) on the Cipres Science Gateway (Miller et al. 2010) using GTR+G+I substitution model with 1000 bootstrap pseudoreplicates. For the BA, substitution models for each locus were estimated using jModelTest-2.1.9 (Guindon and Gascuel 2003, Darriba et al. 2012): in ITS the TIM2ef+I+G, in MCM7 the K80+G, in mtSSU the TPM2uf+I and in nuLSU the TrN+I were used. Two parallel Markov chain Monte Carlo (MCMC) runs were performed in MrBayes 3.2.6 (Huelsenbeck and Ronquist 2001, Ronquist and Huelsenbeck 2003), each using 10,000,000 generations which were sampled every 100 steps. A 50% majority rule consensus tree was generated from the combined sampled trees of both runs after discarding the first 25% as burn-in. Tree files were visualised with FigTree 1.4.2 (Rambaut 2014). The ITS, MCM7, mtSSU and nuLSU sequences are deposited in GenBank (Table 1).

## Results and Discussion

### Phylogeny

**Figure 2.**
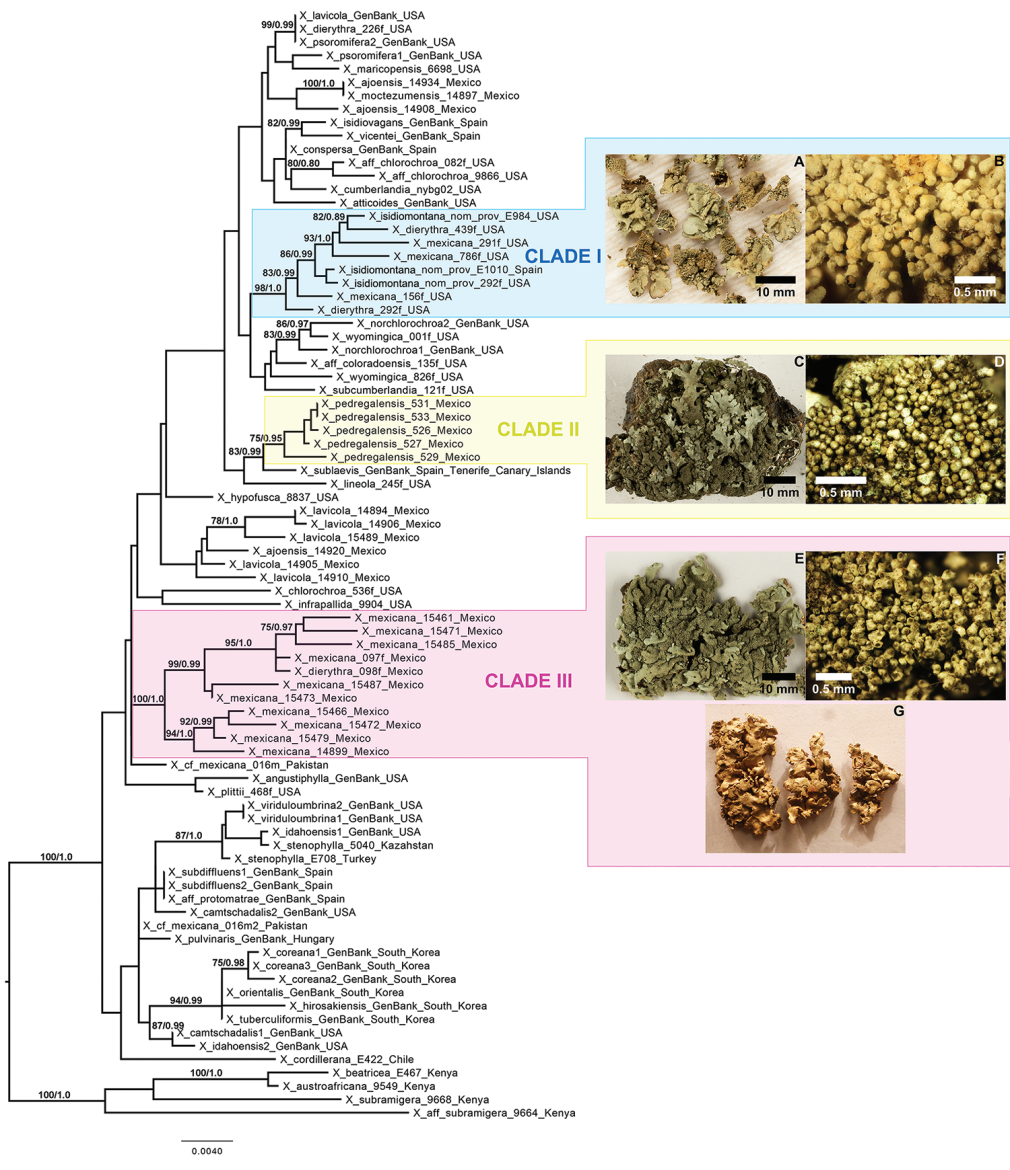
Phylogenetic relationships of the *Xanthoparmeliamexicana* group based on a concatenated data set of ITS, mtSSU, nuLSU and MCM7. Topology based on maximum likelihood (ML) analyses. Bootstrap values above 75 and 0.95 posterior probability are indicated on each branch. The clades I, II and III are highlighted in blue, yellow and pink, respectively. Selected specimens representing clades (habit and isidia): I, *X.mexicana* s. lat. (**A, B**); II, X.*pedregalensis* (**C, D**) and III, *X.mexicana* s. str. (**E, F**), a picture of the *X.mexicana* type specimen from BP is included (**G**).

Results from phylogenetic analyses presented here clearly indicate that the taxonomy in the *Xanthoparmeliamexicana* group requires revision because different samples assigned to the same species based on phenotypical characters may not form a monophyletic group. Specimens identified as *X.mexicana* from Asia (Pakistan and South Korea) were distantly related to samples of the species collected in North America and Europe (included in *X.isidiomontana nom prov*) (Fig. 2). The latter specimens fell into three distinct and well supported clades (clade I-III in Fig. 2). Note that the three distinct and well supported clades did not form a monophyletic group.

Clade ‘I’ (=*X.* ‘*isidiomontana*’ *nom prov*, ‘D2’ in Leavitt et al. 2013) included isidiate specimens from North America and Europe and samples identified as *X.dierythra*, *X.mexicana* (Figs 2A and B) and *X.plittii*, in addition to a number of non-isidiate, fertile specimens. Additional studies will be necessary to better understand the delimitation of *X.dierythra*, which is also polyphyletic and is currently accommodating specimens with norstictic acid and lacking salazinic acid (Hale 1990). This clade likely represents another species-level lineage lacking formal taxonomic recognition and a formal description of this lineage will be proposed once the phylogenetic placement of *X.dierythra* s. str. is ascertained.

Clade ‘II’ included specimens collected in the Pedregal, south of Mexico City, which is also the type locality of *X.mexicana*. However, the new material does not correspond phenotypically with the type specimen of *X.mexicana* in BP (Fig. 2G). These specimens are different from *X.mexicana* specimens (represented by Clade III in phylogenetic analysis) in having less contiguous lobes, densely isidiate thallus, presence of salazinic acid, norstictic acid and an unknown substance. Since clade ‘II’ differs phylogenetically and phenotypically from clade ‘III’ (representing *X.mexicana* s. str. – see below), we describe clade ‘II’ as a species new to science, *X.pedregalensis* (Figs 2C and D).

Clade ‘III’ includes the majority of samples identified as *X.mexicana* collected in different localities of Mexico (Oaxaca, Puebla, San Luis Potosí, Querétaro, Hidalgo). Specimens recovered in this clade were morphologically and chemically similar to the lectotype of *X.mexicana* in BP (Fig. 2G). Therefore, clade ‘III’ is here recognised as *X.mexicana* s. str. (Gyelnik 1931, Hale 1974) (Figs 2E and F). So far, we have only been able to confirm the presence of *X.mexicana* s.str. in Mexico. Specimens collected in other areas and previously identified as *X.mexicana* likely represent different species. For example, none of the samples from Asia or those reported in Leavitt et al. (2013) from western United States belongs to *X.mexicana* s. str. Further studies are needed to evaluate the occurrence of this species in other parts of the world, including North America and Europe.

Underestimates of species diversity is common amongst under-studied organismal groups (Pawar 2003, Chiarucci et al. 2011, Lücking 2012, Coleman 2015, Troia and McManamay 2016, Troudet et al. 2017), which is particularly evident in lichenised fungi (Crespo and Perez-Ortega 2009, Crespo and Lumbsch 2010, Leavitt et al. 2011, Lumbsch and Leavitt 2011, Leavitt et al. 2013, Leavitt et al. 2016, Lücking et al. 2016, Leavitt et al. 2018). Previous studies concluded that the species delimitation in lichenised ascomycetes with traditional morphological and chemical characters are apparently misleading with respect to species diversity. In the study of Leavitt et al. (2016), several new taxa were described primarily based on evidence from genetic data, but it does not preclude the possibility that additional studies investigating morphological and chemical characters may identify additional independent characters or combinations of characters, supporting the species circumscribed using molecular data. Our results corroborate findings from the previous studies by showing the need of an integrative approach using not only conventional (i.e. morphology and TLC data), but also new sets of data (e.g. DNA sequence data) to better understand the pattern of species diversity. Our study shows that, by incorporating molecular data, the taxonomic status of a conventionally difficult group based on morphology can be resolved: the three main clades belonging to the *X.mexicana* complex do not form a monophyletic group based on our newly reconstructed phylogeny (Fig. 1). In this context, the species diversity in the *X.mexicana* complex is likely under-estimated and morphologically cryptic species may be identified in the future.

### Taxonomy

#### Xanthoparmelia pedregalensis

Taxon classificationFungiLecanoralesParmeliaceae

Barcenas-Peña, Lumbsch & Leavitt sp. nov.

MB826958

Figs 2C and D

##### Type.

MEXICO. Ciudad de México: Coyoacán, Pedregal de San Angel, 19°19'8.3"N, 99°11'25.93"W, 2321 m elev., xerophytic scrub, on rocks, November, 2017, Ruiz Cazares 1553 (MEXU-holotype), same locality and date Ruiz Cazares 1559 (MEXU-paratype).

##### Diagnosis.

Thallus moderately adnate to adnate, imbricate, upper surface yellow-green, lower surface tan-brown, abundant isidia subglobose to cylindrical, simple to branched and medulla containing salazinic and norstictic acids as major compounds and an unknown substance. Differing from the phenotypically similar *X.mexicana* by nucleotide position characters in the ITS sequence as shown in Table 2.

**Table 2.** Differences of nucleotide positions in the ITS marker between *X.mexicana* and *X.pedregalensis*.

Species	Aligned nucleotide position characters in the ITS marker							
	36	115	379	425	450	466	488	496
*** X. mexicana ***	G	C	A	C	T	C/T	G	A
*** X. pedregalensis ***	A	T	G	G	C	A	C	G

##### Etymology.

The taxon name is based on its occurrence in the Pedregal de San Angel region of Mexico.

##### Description.

Thallus foliose, moderately adnate to adnate, 2–7 cm in diam., irregularly lobate; lobes subirregular, elongate, plane to subconvex, 1.5–3 mm wide, not lobulate; apices subrotund, smooth, eciliate. Upper surface yellow-green, smooth, shiny, epruinose and emaculate, densely isidiate; isidia initially subglobose, becoming cylindrical to coralloid branched with age, 0.1–0.2 mm in diam., 0.1–0.9 mm tall; tips syncorticate, brown to dark brown, sometimes weakly erumpent; soralia and pustulae absent. Medulla white, with continuous algal layer. Lower surface tan to brown, plane, moderately rhizinate; rhizines pale to dark brown, simple, 0.5–0.9 mm long. Apothecia rare, sessile, 1–2 mm wide, laminal on thallus; disc cinnamon-brown to dark brown; margin smooth, pruina absent; asci: clavate, 8-spored; ascospores hyaline, simple, ellipsoid, 9–10 × 4–5 µm. Pycnidia rare, immersed conidia bifusiform, 5–7 × 1 µm.

##### Secondary metabolites.

Upper cortex K–, C–, KC–, P–; medulla K+ yellow then dark red, KC–, C–, P+ yellow-orange. Upper cortex with usnic acid (major); medulla with salazinic (major) and norstictic acids (submajor) and an unknown substance (minor) (Rf 28–30, brown in daylight after heating, UV brown-dark, yellow halo after heating).

##### Distribution and ecology.

The new species was found in xerophytic scrub vegetation, in Pedregal de San Angel south of Mexico City, growing on volcanic rocks. It is currently known only from the type locality.

##### Notes.

*Xanthoparmeliapedregalensis* is morphological and chemically similar to *X.mexicana.* However, the latter has more contiguous lobes and is less isidiate than *X.pedregalensis*. In addition *X.mexicana* has salazinic (major) and consalazinic acid (minor) and usually norstictic and protocetraric acids (trace) in the medulla, whereas *X.pedregalensis* contains salazinic (major) and norstictic acids (submajor) and an unknown substance. Distinguishing the two species is supported by molecular data.

##### Additional specimens examined.

Mexico. Ciudad de México: Coyoacán, Pedregal de San Angel, 19°19'8.3"N, 99°11'25.93"W, 2321 m elev., xerophytic scrub, on rocks, November, 2017, Ruiz Cazares 1552 (MEXU); 19°19'15.19"N, 99°11'15.22"W, 2311 m, Ruiz Cazares 1555, 1557 (F).

### New state records

***Xanthoparmeliaajoensis* (Nash) Egan, 1975: 217.**

*Parmeliaajoensis* Nash, 1974: 234. [Type collection: Organ Pipe Cactus National Monument, Pima Co., Arizona, USA, Nash 5999 (ASU, holotype; DUKE, US, isotypes).] New to Oaxaca, *X.ajoensis* is distributed across western USA and Mexico where it has previously been reported from Baja California Sur, Durango, Morelos, Puebla, Sinaloa and Sonora on acidic rocks, often in open, arid habitats at relatively low elevations (Hale 1990, Nash and Elix 2004, Nash et al. 2016).

**Specimens Examined**: Mexico. Oaxaca: Quiotepec, 17°54'18.9"N, 96°58'01.8"W, 696 m elev., xerophytic scrub, on rock, October, 2016, Barcenas-Peña 5906, 5908, 5913, 5915 (MEXU).

***Xanthoparmeliamoctezumensis* Nash in C. Culberson, Nash & Johnson, 1979: 155. [Type collection: 28 km E of Moctezuma, Sonora, Mexico, Nash 12548 (ASU, holotype; DUKE, US, isotypes).**]

New to Puebla. *Xanthoparmeliamoctezumensis* is distributed throughout south-western USA and Mexico where it has been reported from Baja California Sur, Durango, Sinaloa and Sonora on acidic rocks, often in open, arid to woodland habitats (Nash and Elix 2004, Nash et al. 2016).

**Specimens Examined**: Mexico. Puebla: San Rafael Coxcatlán, 18°13'16.6"N, 97°07'22.4"W, 1148 m elev., xerophytic scrub, on rock, October, 2016, Barcenas-Peña 5887, 5890, 5891, 5893 (MEXU).

***Xanthoparmeliamexicana* (Gyelnik) Hale, 1974: 488.**

New to Querétaro, San Luis Potosí and Zacatecas. *Xanthoparmeliamexicana* has been reported from Baja California, Baja California Sur, Chihuahua, Coahuila, Distrito Federal, Durango, Guanajuato, Hidalgo, Jalisco, Michoacán, Nuevo León, Oaxaca, Puebla, Sonora and Veracruz, on acidic rocks, often on soil near the coast in open, arid habitats (Nash et al. 2004, 2016).

**Specimens Examined**: Mexico: Querétaro. Tequisquiapan, Rancho Las Fuentes, 20°33'51.0"N, 100°01'54.6"W W, 1942 m elev., xerophytic scrub, on rock, August, 2017, Barcenas-Peña 7516; San Luis Potosí, Mexquitic de Carmona, La Campana, 22°15'28.9"N, 101°05'26.8"W, 2012 m elev., xerophytic scrub, on rock, August, 2017, Barcenas-Peña 7441; Zacatecas, Fresnillo, El Poleo, 23°06'16.4"N, 102°54'24.3"W, 2227 m elev., xerophytic scrub, on rock, August, 2017, Barcenas-Peña 7356 (all MEXU).

## Supplementary Material

XML Treatment for Xanthoparmelia pedregalensis
